# Green and Low-Cost Modified Pisha Sandstone Geopolymer Gel Materials for Ecological Restoration: A Phase Review

**DOI:** 10.3390/gels10050302

**Published:** 2024-04-29

**Authors:** Changming Li, Yubing Fu, Haifeng Cheng, Yaozong Wang, Dongyang Jia, Hui Liu

**Affiliations:** 1 Key Laboratory of Ecological Environment Protection and Restoration in the Yellow River Basin of Henan Province, Zhengzhou 450045, China; tuzi951@163.com; 2 School of Civil Engineering and Transportation, North China University of Water Resource and Electric Power, Zhengzhou 450045, China; dryubingfu@163.com (Y.F.); chf6077@163.com (H.C.); wangyaozong2022@163.com (Y.W.); dongyangjia2022@163.com (D.J.); 3 International Joint Research Lab for Eco-Building Materials and Engineering of Henan, North China University of Water Resources and Electric Power, Zhengzhou 450045, China

**Keywords:** Pisha sandstone, geopolymer gels, physical and chemical characterization, ecological restoration, resource applications

## Abstract

Pisha sandstone (PS) is a special interbedded rock in the middle reaches of the Yellow River that experiences severe weathering and is loose and broken. Due to severe multiple erosion events, the Pisha sandstone region is called “the most severe water loss and soil erosion in the world” and “the ecological cancer of the earth”. As a special pozzolanic mineral, PS has the potential to be used as precursors for the synthesis of green and low-carbon geopolymer gel materials and applied in ecological restoration. This paper aims to undertake a phase review of the precursors for geopolymer gel materials. The genesis and distribution, physical and chemical characterization, erosion characteristics, and advances in the ecological restoration of PS are all summarized. Furthermore, current advances in the use of PS for the synthesis of geopolymer gel materials in terms of mechanical properties and durability are discussed. The production of Pisha sandstone geopolymer gels through the binder jetting technique and 3D printing techniques is prospected. Meanwhile, the prospects for the resource application of PS in mine rehabilitation and sustainable ecology are discussed. In the future, multifactor-driven comprehensive measures should be further investigated in order to achieve ecological restoration of the Pisha sandstone region and promote high-quality development of the Yellow River Basin.

## 1. Introduction

The Yellow River watershed is an important ecological barrier in China. However, the ecological environment of the Yellow River watershed is in a severe situation [1,2]. Soil erosion has become one of the most serious and complex global issues, significantly affecting soil nutrients, land productivity, and carbon cycling in terrestrial ecosystems, and further generating unfavorable environmental and economic impacts [3,4]. At large spatial scales, soil erosion can increase sediment deposition in river channels and downstream reservoirs, thereby increasing the risk of flooding and potential water pollution [5,6]. 

Located in the Yellow River Basin of North China and covering almost 454,000 km^2^, the Loess Plateau is an important ecological barrier and fragile representative region. As the most serious region of soil erosion in China, the Loess Plateau accounts for 98% of the total region of soil erosion in the Yellow River Basin. Furthermore, it contributes 97% of sediment to the Yellow River, which seriously restricts the sustainable development of the Yellow River Basin [7]. The junction region of Shanxi, Shaanxi, and Mongolia in the middle reaches of the Yellow River is the most characteristic erosion region in the Loess Plateau, covering an area of 1.17 × 10^4^ to 3.20 × 10^4^ km^2^ [8,9,10,11,12]. There is a special interbedded rock named Pisha sandstone (PS), which is severely weathered, loose, and broken. PS is as hard as stone when it is dry but rapidly disintegrates into sediment when it is exposed to water. It is the primary source of coarse sediment in the Yellow River. PS mainly refers to the interbedded rock composed of a thick layer of sandstone, mudstone, siltstone, and arenaceous shale in the Paleozoic Permian, Mesozoic Triassic, Jurassic, and Cretaceous. The Pisha sandstone region is characterized by fragmented topography, crisscrossing gullies, low soil nutrients, and low ecological carrying capacity. The climate is characterized by dramatic regional temperature variations, wind and sand, and frequent rainstorms. There are various types of erosion in this region, exhibiting a characteristic of interaction. The soil erosion modulus can reach approximately 30,000–40,000 t/(km^2^·a) [13]. The average annual sediment transport in the Pisha sandstone region is around 200 million t, accounting for about 30% of the total sediment imported in the middle and upper reaches of the Yellow River in the Loess Plateau region [14,15]. The coarse sediment (*d* ≥ 0.05 mm) imported into the Yellow River in the Pisha sandstone region accounts for about 62% of the total coarse sediment imported into the Yellow River [16]. Additionally, around a quarter of the sediment is deposited in the lower reaches, which is one of the ‘culprits’ of frequent floods caused by riverbed uplift [17]. Furthermore, the coexistence of multiple hazards of desertification and soil erosion in the region leads to severe ecological degradation and a decline in carbon sequestration, exacerbating climate warming. Scholars call it “the most soil erosion in the world” and “the ecological cancer of the earth”.

Great efforts have focused on ecological restoration since the 1960s. Significant and encouraging progress has been achieved in the management practice of the Pisha sandstone region through a series of projects such as the National Key Project for Soil and Water Conservation, the Soil and Water Conservation Project on the Loess Plateau supported by a World Bank loan, and the Key Project for the Management of Small Watersheds. The ecological restoration in the Pisha sandstone region is facing new opportunities and challenges, with the proposal of the national strategy of “Ecological Protection and High-Quality Development in the Yellow River Basin” [18]. PS has good pozzolanic activity due to its high contents of silica and alumina. There is an excellent opportunity to develop PS as a green, low-carbon geopolymer gel material to control soil erosion and restore ecology. At the same time, based on Pisha sandstone geopolymer gel materials, the material preparation process can be developed to meet different engineering purposes. Combined with the potential ecological restoration function of Pisha sandstone, the development of comprehensive measures for ecological restoration is a future research direction.

Therefore, it is necessary to carry out a phased review to facilitate a comprehensive understanding of Pisha sandstone as well as the ecological restoration techniques, and to demonstrate the main problems and gaps faced by ecological restoration in the Pisha sandstone region. This paper reviews the current studies related to the physical and chemical properties, erosion characteristics, ecological restoration advances, and potential applications of PS, with particular emphasis on the properties of modified Pisha sandstone geopolymer gel materials. Additionally, prospects for the potential applications of PS in mining area restoration are also reviewed. Finally, the future direction of ecological restoration is prospected with new insights to establish a multifactor-driven comprehensive technical model. 

## 2. Distribution of Pisha Sandstone

In the Paleozoic before the Carboniferous, the Pisha sandstone region was a sea. During the Permian period, the crust was uplifted and the sea disappeared, turning into an inland basin where red mudstone and clastic rock of continental facies were deposited. In the early and middle Triassic, the basin sank rapidly, and red mudstone and clastic rock of continental facies continued to be deposited. Subsequently, the basin was uplifted under the influence of Indonesian tectonic movement. Weathering erosion caused false conformity and angular unconformity contact between strata. In the late period, the crust slowly descended, causing parallel and angular unconformity contacts between strata. Meanwhile, the red stratum gradually decreased, and organic substances such as gray-green clastic rocks and coal-bed lenses began to be deposited, which was characterized by lacustrine deposition. At the end of the period, due to the Indochinese tectonic movement, the basin was fully uplifted and the strata were generally eroded. In the early Jurassic period, the crust gradually sank. The climate changed from a dry and hot oxidation environment to a warm and humid reduction environment. Meanwhile, gray-green and gray coal-bearing fine sand shale strata began to be deposited. In the middle and late Jurassic, due to the descent of the crust, the climate transitioned from warm and humid to dry and hot. Meanwhile, red mudstone deposits appeared. Under the influence of the late Yanshan tectonic movement, the crust continued to rise. The basin was uplifted for a long time and suffered from weathering and stripping. Meanwhile, the Lower Cretaceous was unconformity over the Middle Jurassic. In the Cretaceous, the crust turned to sink, and mudstone and coarse debris were deposited in large amounts, which were characterized by lacustrine deposition. At the end of the Cretaceous, the crust was gradually lifted by the late Yanshan tectonic movement, and the uplifted basin suffered wind erosion. In the Pliocene period of the Tertiary, the crust began to sink, and the red mudstone clay layer was deposited, which was characterized by lacustrine deposition. At the end of the last century, a plateau was formed during the upward and downward movements of the crustal structure. In the Quaternary period, the loess became sand, ancient humans appeared, and the modern landscape was formed [19,20,21,22,23,24]. 

**Figure 1:**
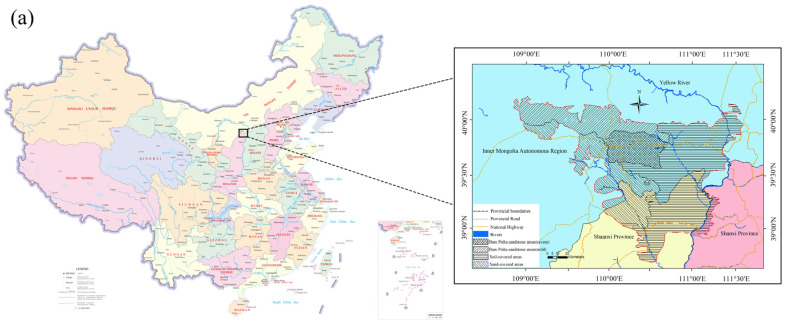

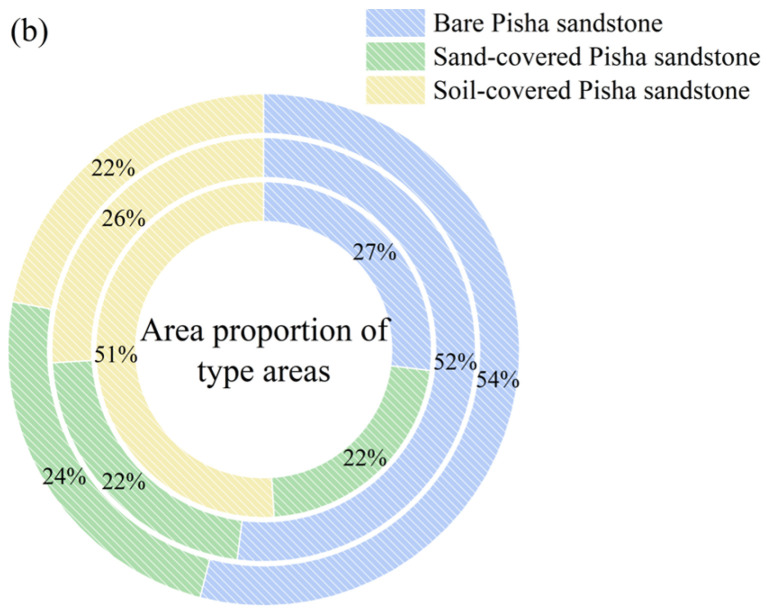
The distribution of Pisha sandstone. (**a**) The distribution map of Pisha sandstone in China [8]. (**b**) Proportional area of Pisha sandstone-type regions [8,9,10].

At present, PS is concentrated in the junction region of Shanxi, Shaanxi, and Mongolia, ranging from the Yellow River in the east, reaching Maobula River in Hangjin Banner of Inner Mongolia in the west, Shenmu County in Shaanxi Province in the south, and the southern edge of Hobq Desert in the north. Erdos Dongsheng District, Junger Banner, Ejin Horo Banner, Dalad Banner, and Hangjin Banner are the main distribution region of PS. There are scattered distributions in Shenmu County and Fugu County in Shaanxi, Hequ County and Baode County in Shanxi, and Qingshui River County in Inner Mongolia [8]. The distribution of Pisha sandstone is shown in Figure 1. Depending on the surface cover, the rocky region can be divided into three types of regions: bare PS region, sand-covered PS region, and soil-covered PS region. The distribution region of PS is shown in Table 1. It can be seen that the distribution region of PS ranges from 1.17 × 10^4^ to 3.2 × 10^4^ km^2^. The defined regions may vary due to different research methods and definitions of the type of regions, as well as the fact that the bare PS near the desert may evolve into sand-covered PS over time. 

## 3. Physical and Chemical Characterization of Precursors for Pisha Sandstone Geopolymer Gels

### 3.1. Physical and Chemical Properties

**Figure 2:**
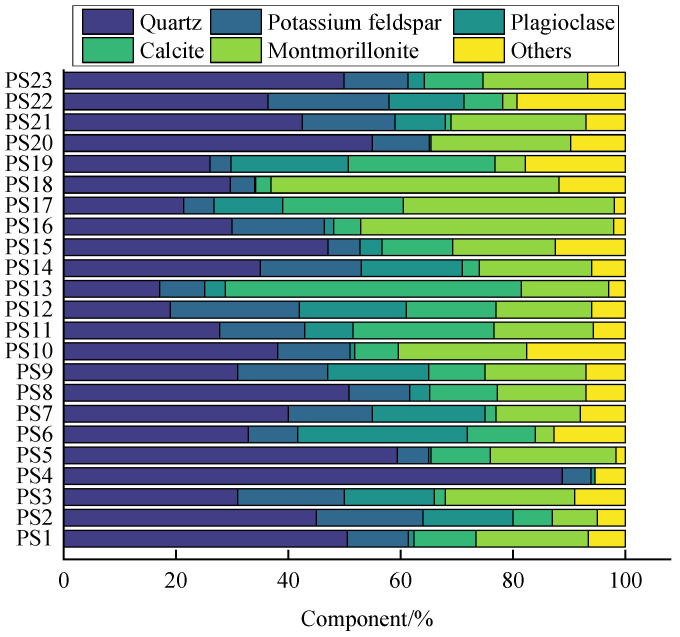
Mineral composition of PS [11,28,29,30,31,32,33]. Note: PS1–7 are gray-white, PS8–11 are purple-red, PS12–15 are pink, PS16–19 are yellow, PS20 is gray-green, and PS21 is gray-white and purple-red stripes. PS2, PS3, PS9, and PS12 are cited from the literature [11]; PS1, PS8, and PS21 are cited from the literature [28]; PS4-6, PS10, PS11, PS13, and PS16–20 are cited from the literature [29]; PS23 is cited from the literature [30]; PS7 and PS14 are cited from the literature [31]; PS15 is cited from the literature [32]; and PS22 is cited from the literature [33].

PS shows a diversity of colors due to its different strata and environment. The main colors are pink, purple-red, gray-white, gray-green, and yellow-green [16,25,26,27]. The mineral composition of PS of different colors and regions is shown in Figure 2 [11,28,29,30,31,32,33]. It can be seen that PS is mainly composed of quartz (17.10% to 88.80%), calcium montmorillonite (2.51% to 51.33%), potassium feldspar (3.70% to 23.00%), plagioclase (0.20% to 30.20%), calcite (1.00% to 52.70%), illite (0.22% to 22.00%), kaolinite (0.00% to 44.00%), etc. In addition, some PS contains small amounts of hematite, with a content of about 1%. In addition, mica minerals are also detected in PS [33]. The contents of plagioclase, calcite, illite, and kaolinite vary considerably for different regions of PS. The contents of quartz, calcium montmorillonite, plagioclase, and calcite vary greatly among different colors of PS. However, there is little difference in other mineral contents. The large amount of quartz in the Pisha sandstone can provide particle support for geopolymer gels. Meanwhile, clay minerals such as calcium montmorillonite, kaolinite, and illite offer the possibility of Pisha sandstone as a reactive precursor for geopolymer gels.

**Figure 3:**
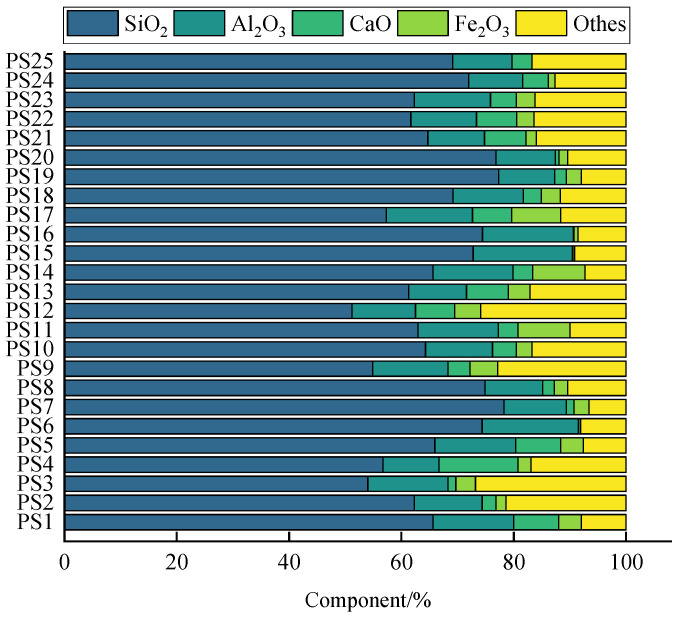
Chemical component of Pisha sandstone [9,11,26,27,30,32,34,35,36,37,38,39,40]. Note: PS1–7 are gray-white, PS8 and PS9 are purple, PS10–15 are pink, and PS16 is yellow. PS22 is cited from the literature [9]; PS2, PS3, PS8, and PS11 are cited from the literature [11]; PS4, PS9, and PS12 are cited from the literature [27]; PS6 and PS14 are cited from the literature [35]; PS5 and PS13 are cited from the literature [36]; PS17 is cited from the literature [30]; PS18 is cited from the literature [32]; PS1 and PS10 are cited from the literature [38]; PS19–21 are cited from the literature [34]; and PS7, PS15, and PS16 are cited from the literature [39].

The chemical component of PS is shown in Figure 3 [9,11,26,27,30,32,34,35,36,37,38,39,40]. It can be seen that the main chemical components of PS are SiO_2_ (51.20~78.25%), Al_2_O_3_ (9.57~15.37%), and Fe_2_O_3_ (0.05~9.28%). The total contents of alkaline oxides Na_2_O, K_2_O, and CaO are 0.90~20.76%. The contents of other chemical components are lower, within 0.00~1.00%. The presence of alkaline oxides makes the rocks alkaline, with pH values ranging from 7.66 to 10.02. The difference in the content of the main chemical components of PS of different colors is small. The chemical components with a low content, such as CaO and Fe_2_O_3_, have greater differences in content. The content of CaO in gray-white PS is the highest, exceeding 14.00%. The content of Fe_2_O_3_ in red PS is about 9.00%. The difference in Fe_2_O_3_ content is the main reason for the red and white color of PS. In addition, some PS also has small amounts of SO_3_, CO_2_, MnO, and P_2_O_5_. The abundance of silica–aluminum–calcium chemicals in Pisha sandstone provides a source for the formation of Pisha sandstone geopolymer gels. However, the activity of these chemicals determines the amount of geopolymer gel produced. The method of excitation of the activity needs to be further investigated.

**Figure 4:**
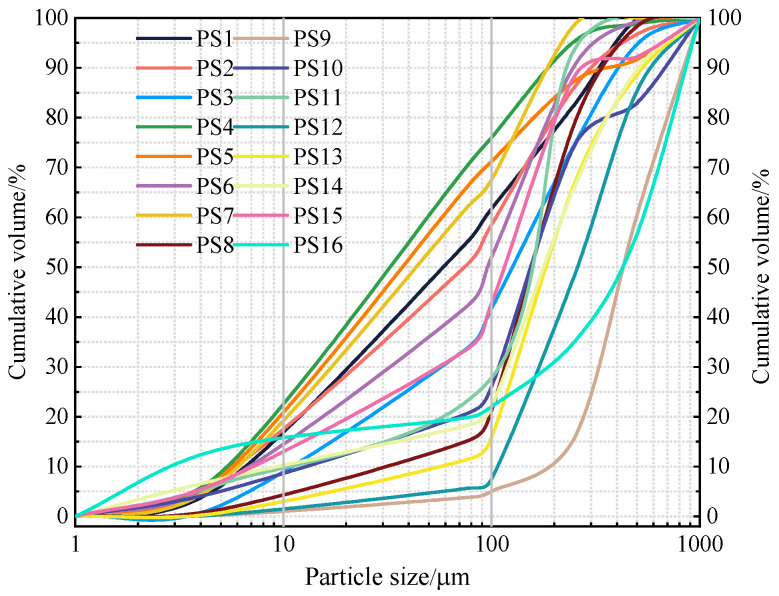
Cumulative distribution curves of PS. Note: PS 1–6 are cited from the literature [26], PS 7 and PS 8 are cited from the literature [31], PS 9 is cited from the literature [35], PS 10 and PS 11 are cited from the literature [40], PS 12 is cited from the literature [41], PS 13 is cited from the literature [45], PS 14 and PS 15 are cited from the literature [46], and PS 16 is cited from the literature [47].

In general, the bulk density of PS is 1.56–2.50 g/cm^3^, and the particle density is 2.62–2.84 g/cm^3^. The porosity is 6.02~35.15%. The permeability coefficient is 5.2 × 10^−3^ mm/s. The liquid limit *W*_L_ and plastic limit *W*_p_ are 29.30% and 19.60%, respectively. The plasticity index *I_p_* is 9.40 [38,41,42,43]. This indicates that the Pisha sandstone has large pores and good permeability, and that the rock structure is loose. The cumulative distribution curves of the particle size of PS are shown in Figure 4. It can be seen that the rock particles mainly consist of 10.73~57.60% of medium and fine sand (0.10~0.25 mm) and 1.40~44.76% of medium and coarse sand (0.25~0.50 mm). Most of the particles are concentrated in the range of 0.10~0.50 mm, accounting for 50.00% of the total. The percentage of clay particles with *d* ≤ 0.005 mm is 1.40~10.40%. The coefficient of nonuniformity of PS ranged from 2.55% to 23.90%, which is poorly graded sand. The curvature coefficient is 0.51~5.54%. The content of sand and powder particles is large, and the content of clay particles is relatively small. Therefore, PS is a typical sandy soil and has a non-equigranularity structure [26,28,30,31,35,38,40,41,42,43,44,45,46,47].

The internal pores of PS are numerous and poorly connected, indicating that the distribution is non-homogeneous. Mineral particles are mainly connected by cementation. The types of cementations are divided into pressure-embedded cementation, substrate-porous cementation, and crystal stock-contact cementation. Carbonate and clay are the main cement. Pressure-embedded cementation structures are very tightly cemented between particles, with fewer pores and fissures between particles, which are mainly filled and wrapped by muddy cement and flaky cement. The particles of substrate-porous cementation are mainly composed of quartz and feldspar, with fewer inter-particle pores but relatively more cracks. At the same time, there is more cement between the particles and on the surface of the particles. The particles of the crystal stock-contact cementation structure are mostly adhered by muddy cement, and the degree of cementation between particles is relatively weak [28,38,48]. In conclusion, the cement between the particles of the Pisha sandstone is mainly clay minerals, which can help with the preparation of geopolymer gels. However, the clay mineral content, morphology, and distribution characteristics vary between different-colored Pisha sandstone. It is necessary to combine the respective properties for the synthesis of geopolymer gels from Pisha sandstone.

The mechanical properties of PS are shown in Table 2. The mechanical strength of Pisha sandstone varies according to color. There are also significant differences in the strength of the rock in different states. The compressive strength in the natural state is 0.40 to 39.70 MPa. The compressive strength in the dry state can be increased to 25.30 MPa. The difference in shear strength between the dry and saturated states is small, ranging from 0 to 4.16 MPa. The tensile strength ranges from 0.01 to 0.74 MPa. The compressive strength, tensile strength, and shear strength decrease significantly after encountering water, and the softening coefficient ranges from 0.05 to 0.42. The low water-saturated shear strength and dry shear strength determine the weak resistance of PS to runoff erosion and collapse [28,30,32,38,40,42,49]. Therefore, water resistance is a factor that must be considered for the application of Pisha sandstone geopolymer materials for ecological restoration.

### 3.2. Erosion Characteristics

**Figure 5:**
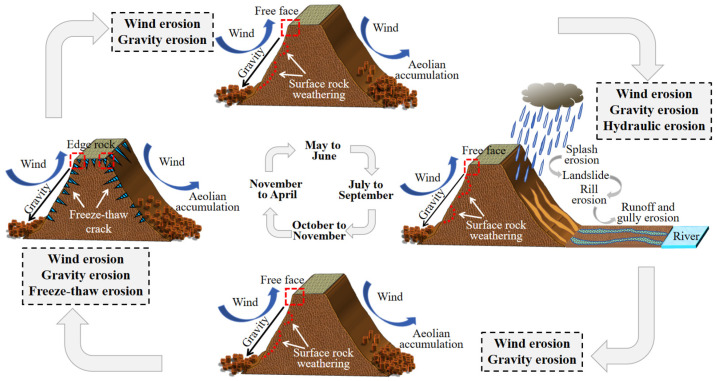
Multiple dynamic erosion cycles on an annual scale.

The Pisha sandstone region is characterized by the interaction of hydraulic erosion, wind erosion, freeze–thaw erosion, gravity erosion, and anthropogenic erosion. Bi et al. [50] classified rock erosion into “seasonal rainfall runoff erosion” and “perennial non-runoff erosion” according to the type of dynamics. Hydraulic erosion occurs in the form of raindrop splashes, slope, and gully erosion. In particular, gully erosion plays an important role in sediment transport, which accounts for more than 70% of the sediment in the basin. The wind direction in the Pisha sandstone region is mostly perpendicular to the gully or crosses the gully at a large angle, which leads to frequent wind erosion. It is characterized by wind accumulation and wind erosion [51]. The high-intensity wind erosion regions are formed between high landforms or windward slopes and both sides. The weak wind erosion regions are formed on leeward slopes or in relatively flat regions. Sediment accumulation regions form on leeward slopes, depressions, and gullies [51,52]. Gravity erosion forms mainly in gullies, caused by weathering and other factors [44]. According to statistics, the sediment yield of gravity erosion accounts for about one third of the total sediment yield in the basin. The main manifestations of gravity erosion are debris slides and landslides. Additionally, the accumulation of various kinds of sliding sandstone blocks or granules caused by slumps and avalanches at the foot of the slope is also an important manifestation of gravity erosion. Furthermore, sliding sandstone blocks would be scoured and disintegrated into coarse sediment in the gradual convergence of the water flow, which indirectly provides a sand source [51]. Freeze–thaw erosion is caused by mechanical damage such as severe temperature fluctuation, solid–liquid circulation transformation of water, and the expansion, contraction, and fracture of rocks. Eventually, the rock moves and is lost under the action of hydraulic and gravity erosion. Furthermore, the bonded water between the particles is transformed into solid water during the freezing process, leading to soil expansion. Subsequently, the melting process of solid water between particles leads to changes in the soil structure, resulting in gravity erosion of marginal soil [53,54]. The multiple dynamic erosion cycles on an annual scale are presented in Figure 5.

The erosion of different types of regions is shown in Table 3 [25,55]. It can be seen that gravity erosion exists in the multiple erosion of all three types of regions. Moreover, freeze–thaw erosion occurs wherever PS is bare. Meanwhile, multiple erosion patterns in the Pisha sandstone region are coupled in space and alternated in time. Sediment loss mainly occurs in summer and autumn. In winter and spring, it is mainly freeze–thaw erosion and hydraulic erosion caused by gravity erosion and wind erosion. The transition period of spring and summer is the high incidence of freeze–thaw erosion and wind erosion. April and May have the high incidence of wind erosion, and May and June have the high incidence of gravity erosion. July to September have the high incidence of hydraulic erosion. May to September are the coupling period of gravity erosion and hydraulic erosion. Other periods are characterized by interactive coupling of wind, gravity, and freeze–thaw erosion [53,56].

## 4. Advances in the Ecological Restoration of Pisha Sandstone Region

In the early 1990s, China began to conduct systematic research on the erosion of PS, and the Yellow River Conservancy Commission of the Ministry of Water Resources completed the “Distribution range of PS and erosion type region in the junction region of Shanxi, Shaanxi and Mongolia” and the “General report of the experimental research stage of plant ‘flexible dam’ in Pisha sandstone region (1995–1998)” [57]. Subsequently, the biological measures mainly based on sea buckthorn were explored through the “National Key Management Project of Soil and Water Conservation”, “Soil and Water Conservation Ditch Control Backbone Project”, “Sea Buckthorn Ecological Project in Pisha Sandstone of Shanxi, Shaanxi and Mongolia”, “Comprehensive Management Project of Small Watersheds”, and “Small Watershed Comprehensive Management Project” [58,59,60]. Good ecological benefits have been achieved. Based on the harness of seabuckthorn, Bi [61] proposed the idea of “a seabuckthorn flexible dam”. Many scholars have successively researched the use of seabuckthorn to construct flexible dams in gully channels to intercept sediment, and many results have been achieved [62,63,64]. However, seabuckthorn showed a phenomenon of withering and death and could only be planted in gullies. Moreover, erosion occurring on slopes and gully slopes was difficult to prevent.

The Institute of Geographical Sciences and Natural Resources Research, CAS, joined enterprises and universities to develop PS and sand compounded into soil technology. Based on the complementary structural characteristics, PS and sand were compounded into a new type of soil, which could help with the resource utilization of PS [65,66]. However, this technology was mainly used for sand transformation, and it was difficult to solve the problem of soil erosion in the Pisha sandstone region. Wen et al. [67,68] found that PS had outstanding electrostatic adsorption and ion exchange capacity and had good adsorption properties, which could realize the adsorption of heavy metals to restore contaminated soil. It was a good natural adsorption material and was economical and environmentally friendly. Zhen et al. [69] selected three types of typical soils in mining areas, namely, PS, loess, and sandy soil, and designed the soil structure to simulate the infiltration process. It showed that PS could reduce the infiltration capacity of soil and could be applied to the ecological restoration of mining areas. Su et al. [70,71] carried out experiments on the effect of EN-1 curing agent on the mechanical properties of PS. The hydrodynamic properties under different factors were investigated by simulating the slope runoff scour. The optimum curing agent dosage, compaction, moisture content, and curing age were obtained. However, the current EN-1 curing agent could not be directly used as a modified material, because it could not meet the comprehensive performance requirements of consolidation, erosion resistance, water storage, and vegetation promotion. Meanwhile, it could not cope with the environment of multiple erosion coupling and interaction. It was difficult to solve the erosion problem of high slopes. Based on the project “Integration and Demonstration of Anti-erosion and Vegetation-promoting Technology in Pisha Sandstone Region in the Middle Reach of the Yellow River”, the Yellow River Institute of Hydraulic Research has considered the limitations of traditional engineering measures and biological measures and invented anti-erosion and vegetation-promoting materials and modified dam-building technology. Meanwhile, soil and water conservation material, engineering biological measures, and a two-dimensional slope–gully integrated management mode system were proposed, and the management effect was remarkable [72,73,74,75]. Meanwhile, the Dalian University of Technology carried out research on modified dam construction technology and prepared modified dam construction materials with good performance [35,36,76].

**Figure 6:**
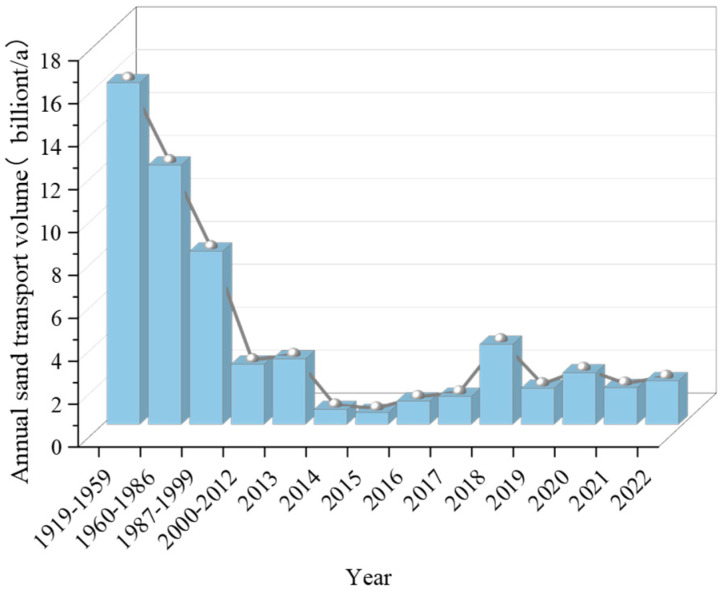
Annual sediment transport in the Yellow River [77].

Figure 6 presents the trend of sediment delivery from the Yellow River over the last 100 years [77]. The water and soil conservation work in the middle reaches of the Yellow River has been effective in recent years. It can be seen that the measured annual sediment transport at Tongguan station in 2021 was 171 million t, which showed a significant decrease compared to the average annual sediment transport of 1.592 billion t from 1919 to 1959. However, the contradiction of the incongruent water–sediment relationship was still prominent. Focusing on the Pisha sandstone region, there were deficiencies in the comprehensiveness, uniformity, and continuity of the implementation of traditional harness measures. Moreover, there was a lack of integrated and comprehensive region-wide and mechanism-wide harnesses. The trend of ecological deterioration in the Pisha sandstone region has not been fundamentally curbed.

## 5. The Synthesis of Pisha Sandstone Geopolymer Gels

As a special pozzolanic mineral, Pisha sandstone contains large amounts of clay minerals such as calcium montmorillonite, kaolinite, and illite. Alkali activation and thermal activation can turn clay minerals into a highly reactive precursor, providing a source for the synthesis of geopolymer gels. Thermal activation causes dehydroxylation of the clay minerals, resulting in the formation of high-energy chemical bonds that make it very readily available for reaction. Alkali activation dissolves the silicate monomers in the clay mineral, providing a source of reactants for the polycondensation reaction [78,79]. The effects of saturated lime water and different concentrations of sodium hydroxide solution on the dissolution of SiO_2_ and Al_2_O_3_ in PS were also studied. It was concluded that the dissolution of SiO_2_ and Al_2_O_3_ in Pisha sandstone increased significantly with the concentration of NaOH solution. The dissolved amount reached above 30% in 2 mol/L NaOH solution, showing high activity. At the same time, the activity of PS was studied by a mortar strength test. The compressive strength results of the collodion specimens showed that the activity index of arsenopyrite was close to 60%, and the flexural strength test results showed that the activity index of arsenopyrite was around 65% [35].

Considering that the mechanical properties of the material were not excellent when only PS was used as a precursor, additional sources of precursors were combined to generate stronger geopolymer gels [80,81]. Pisha sandstone combined with mineral additives was used as a raw material, and alkaline modifiers were used to stimulate mineral activity to prepare geopolymer gel materials. Specimens were prepared by using a compression molding method for ambient- or high-temperature curing. The mechanical and microscopic properties of the materials were investigated by compressive strength tests and microscopic tests (XRD, TG, FTIR, SEM, EDS). Combined with the results of microscopic tests, the significant improvement in the mechanical and durability properties of the material was attributed to the generation of a large number of geopolymer gels that bound the particles to form a stable skeletal structure. PS–fly ash composites were prepared with a maximum compressive strength of 20.3 MPa under optimal conditions. It was concluded that the main factors affecting the mechanical properties of the material are the fineness of the PS, type of activator, fly ash amount, alkali content, and curing temperature. PS was also calcined to prepare PS–fly ash composites. The results showed that the thermal activation treatment improved the reaction level and enhanced the mechanical properties of the materials. The compressive strength was up to 21.9 MPa. In addition, the water resistance and permeability of the PS–fly ash geopolymer materials were also studied. With the increase in the amount of fly ash, the total porosity of the materials decreased, the water permeability resistance increased significantly, and the softening coefficient reached 0.86 [82,83,84,85]. The application potential of blast furnace slag–PS composites, steel slag–PS composites, and silica fume–PS composites was also studied. The results showed that the compressive strength of blast furnace slag–PS composites and steel slag–PS composites reached up to 56.2 MPa and 46 MPa, respectively, under the optimum level of mineral replacement (40%) and optimum activator (1.5% NaOH). The compressive strength of silica fume–PS composites reached 44.8 MPa with the addition of 10% silica fume and 3% sodium hydroxide. Meanwhile, blast furnace slag–PS composites and silica fume–PS composites had excellent water resistance with softening coefficients of 0.95 and 0.98, respectively [86,87,88].

PS-based cement gel materials have also been widely studied. Dong et al. [36] studied the feasibility of PS as a concrete admixture. The results showed that the flexural strength and durability of the material increased significantly when the amount of PS was lower than 20%. The initial setting time and compressive strength were not affected. However, the performance of the material decreased significantly when the amount exceeded 30%. Geng et al. [89] studied the effect of cement amount on the properties of PS. The results showed that with the increase in cement incorporation, the porosity of PS gradually decreased, the cohesion tended to increase, the material tended to be brittle, the pore deterioration of PS under freeze–thaw action was reduced, and the frost resistance was significantly improved. It was concluded that the optimum cement amount was 20%. Dong et al. [90] selected sodium hexametaphosphate and calcium lignosulfonate as plasticizers. The mechanical properties, rheological properties, dry shrinkage properties, and hydration properties of PS–cement composites were investigated. It was concluded that the compressive strength of the material reached 52.7 MPa after curing at 80 °C under the optimal mixing ratio. Yang et al. [91,92] studied the effects of fly ash and basalt fiber on the mechanical properties of PS–cement materials. It was concluded that a small amount of fly ash improved the compactness and deformation control of the material. Basalt fibers with a content of less than 0.2% had a reinforcement effect on the material, which inhibited the deformation of the material. Zhang et al. [93,94] tested the performance of cement mortar with PS as a substitute for river sand. The results showed that the substitution rate of red PS below 10% had little effect on the dry shrinkage of specimens at various ages. Concrete of different strength grades met the mechanical property requirements at a 20% substitution rate. However, the water–cement ratio of cement mortar increased significantly at a substitution rate of more than 50%. This led to a decrease in strength and an increase in dry shrinkage, resulting in poor durability. Therefore, the addition of a water reducer to control the water–cement ratio was proposed to improve the substitution rate of PS and the mechanical properties of cement mortar. The feasibility of replacing river sand with PS in the preparation of building materials was verified.

## 6. Ecological Restoration Applications of Pisha Sandstone Geopolymer Gels

### 6.1. Engineering Composite Materials

**Figure 7:**
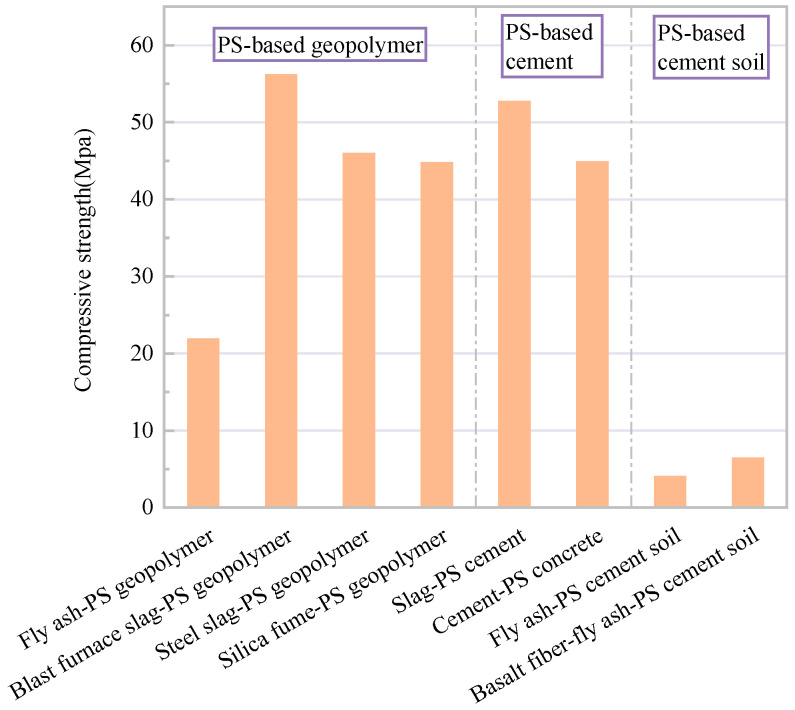
Compressive strength of different types of engineering composite materials for Pisha sandstone.

The most common and conventional approaches are engineering measures to control water and soil loss. Engineering measures include slope engineering (horizontal ditch, fish-scale pit, ditch edge ridge, intercepting ditch, etc.), ditch head protection engineering, and ditch engineering (check dam, pond dam, small retaining engineering, etc.). However, Pisha sandstone cannot be used as an engineering material directly because of its poor cementation and poor anti-water erosion ability. Industrial wastes such as PS, fly ash, blast furnace slag, silica fume, and steel slag are rich in silicate minerals. These minerals are more reactive under alkali activation and can produce more compact hydration products, resulting in a more stable backbone structure and producing engineering composite materials with even better properties [95,96]. Figure 7 presents the mechanical properties of different types of engineering composite materials for Pisha sandstone. The mechanical properties of the composite materials prepared from Pisha sandstone combined with solid waste (e.g., blast furnace slag, fly ash, silica fume, steel slag) meet the engineering requirements. Moreover, the composite materials have excellent resistance to water erosion [82,83,84]. This can be attributed to the fact that the generated geopolymer gels fill the pores of the material and the particles are more tightly bonded to form a stable skeleton structure [80]. Based on the above research, materials for dam engineering were prepared and applied in the Erlaohuo gully dam project of Ordos Jungar Banner to verify the feasibility of PS-modified materials in the engineering of check dams in Pisha sandstone regions [72,97].

On the other hand, PS-based cement materials used in the construction of check dams and slope engineering have also been widely studied [36,89,93,94]. However, even though concrete has desirable mechanical strength, there are limitations to its durability performance. Due to its poor deformation control, Pisha sandstone expands and contracts after the freeze–thaw process, resulting in cracking and damage to the concrete. Fly ash and basalt fibers have a reinforcing effect on the material and can improve deformability [92,98]. Nevertheless, further research is necessary on the durability properties of PS-based cement materials. In addition, Zhu et al. [91,99] prepared compound soil by mixing fly ash with PS cement soil through alkali excitation technology. It was concluded that fly ash significantly improved the mechanical properties and microstructure of PS cement soil. The strength of the compound soil reached 6.85 MPa, which met the design requirements of highway structural soil.

However, Pisha sandstone geopolymer gel materials have limitations in practical applications. The preparation process of the material is unable to cope with different functional requirements for ecological restoration. There is an increasing trend in 3D concrete printing technology. PS-based geopolymer material and PS-based cement material are improved to be binder jetting materials applied to slope protection projects and in ground dam projects, which will greatly improve the operability and application of ecological restoration projects. At the same time, the material preparation process is upgraded to 3D printing technology, which makes the material both producible and economical [100,101,102]. This is a potential research direction for the future. Ensuring the strength of the slurry while controlling the fluidity of the sprayed slurry is the key technical issue. Meanwhile, the curing process of printed blocks is also an issue worthy of in-depth study in the future.

### 6.2. Mine Restoration Green Materials

The Pisha sandstone region is located in the core region of China’s coal energy base. Energy exploitation has brought great economic benefits, but it has also seriously polluted the local ecological environment. In particular, the open-pit coal mine would cause irreversible damage to the atmosphere, soil, vegetation, water source, and topography [103]. Therefore, the ecosystems of coal mine dumps are in urgent need of restoration.

The structure of PS determines its strong impermeability and adsorption [104]. PS could effectively reduce the infiltration capacity of the soil and improve the water-holding capacity. Meanwhile, it could reduce soil toxicity and improve soil physicochemical properties, which have a significant effect on the improvement of polluted soil. Compared to other materials, PS is widely available and can be locally sourced, which has the potential for soil ecological remediation. Ma et al. [105] mixed PS with sandy soil, irrigated silt soil, and loess in different proportions to study the process of water infiltration into the soil. The results showed that the PS significantly reduced the infiltration rate and saturated hydraulic conductivity of the soil. The sandy soil mixed with PS at a ratio of 1:3 had the best water-holding capacity. Zhen et al. [69] selected three types of typical soils in the mine region, namely, PS, loess, and sandy soil, and simulated the infiltration process of single configuration, layered configuration, and soil-rock mixed configuration. Wen et al. [67,68] studied the adsorption effect of three different colors (red, white, and grey) of PS on Pb and analyzed the effects of adsorbent dosage, pH, and ionic strength. The effect of the amount of PS on the morphology distribution and toxic leaching content of Pb was studied through simulation experiments. The changes in soil porosity, soil water-holding capacity, soil enzyme activity, and pH were also studied. The results showed that PS significantly reduced the content of weak acid extractable fraction and toxic leaching in soil. The adsorption effect was mainly related to the Pb concentration, adsorbent dosage, pH, and ionic strength, which was proportional to the adsorbent dosage and pH and inversely proportional to the ionic concentration. In addition, the adsorption rates of different colors of PS were different, showing that gray > white > red.

However, relying only on the impermeability and adsorption properties of the Pisha sandstone protolith to repair the soil is limiting for the restoration of the mine site and cannot deal with the multiple ecological problems in the mine site. The application of the adsorption of materials to solve the problem of heavy metal pollution of industrial wastewater from mining areas has been widely discussed [106,107]. The preparation of binder jetting materials by combining Pisha sandstone with mine wastes to solve the problem of heavy metal ions infiltration underground from mine effluent may be a very interesting research direction. However, the pH has a large influence on the adsorption property because it is closely related to the mechanism involved in the adsorption process. How to control the pH of the material so that Pisha sandstone maintains good adsorption properties is a technical problem that needs to be further studied.

## 7. Conclusions and Prospects

Although many studies and practices have been carried out on ecological restoration in the Pisha sandstone region, further research and development of more innovative and practical technologies should be undertaken. The preparation process of Pisha sandstone geopolymer materials needs to be further improved. Combined with 3D printing technology, preparation as slurry material or extrusion-based block material is a potential future research direction. PS is used as a precursor to synthesize a green and low-loss geopolymer gel material, reducing its negative environmental effects while enhancing ecological sustainability. Additionally, the multi-dimensional harness mode combining modified Pisha sandstone geopolymer gel materials with anti-erosion and vegetation-promoting materials and vegetation measures has good effects on sediment interception, soil erosion retardation, and vegetation restoration, which is the new direction of ecological restoration in the Pisha sandstone region in the future. Furthermore, based on the study of physical and chemical properties, the development of PS into mine restoration materials is also a practical method of resource application. A multifactor-driven synergistic mechanism between resource applications and ecological security should be established to deal with soil erosion, forming a technical model for resource applications, ecological restoration, and security assurance. In the future, multifactor-driven comprehensive technical measures should be further investigated in order to achieve ecological restoration of the Pisha sandstone region and promote high-quality development of the Yellow River Basin.

## Figures and Tables

**Table 1:** Distribution regions of PS unit: 10^4^ km^2^.

Reference	Bare PS	Sand-Covered PS	Soil-Covered PS	Total
[8]	0.45	0.37	0.84	1.66
[9]	0.63	0.26	0.31	1.20
[10]	0.63	0.28	0.26	1.17
[11]	/	/	/	3.20
[12]	/	/	/	1.97 ^①^

Note: ①—The particle size of the sediment yield is greater than or equal to 0.05 mm and the sediment transport modulus is greater than 5000 t/(km^2^·a).

**Table 2:** Mechanical properties of PS.

Reference	Sample Collection Site	Type	Softening Coefficient	Compressive Strength/MPa	Shear Strength/MPa	Tensile Strength/MPa
[28,32]	Jungar Banner, Ordos	Gray-white, purple-red, gray-white with purple-red stripes	0.05~0.42	5.19~39.70 (dry)	0.09~4.15	0.06~0.74
				18.72 (AVG)		
			0.20 (AVG)	1.00~8.69 (water-saturated) 3.73 (AVG)	1.16 (AVG)	0.21 (AVG)
[30]	Boldonggou, Shagedu town, Ordos	Gray-white, purple-red, gray-white with purple-red stripes	-	-	0.090~0.470 (dry)	-
[38]	Erlaohuo gully, Shagedu town, Ordos	White	-	0.40~1.20	-	-
		Red	-	0.90~2.80	-	-
[40]	Jungar Banner, Ordos	Brown red, light red	-	-	0.072 (cohesive force)	-
[42]	Jungar Banner, Ordos	Gray-white with purple-red stripes	-	7.30~10.90	0.0021~0.0082	0.0100~0.0430
		Gray-white	-	13.70~20.10	0.0122~0.0210	0.0527~0.0896
		Purple-red	-	23.20~27.60	0.0275~0.0393	0.1010~0.1696
		Pink	-	30.30~30.60	0.0464~0.0674	0.2275~0.2627
[49]	Jungar Banner, Ordos	-	-	1.33~3.11	-	0.01~0.16

**Table 3:** Erosion modulus of different Pisha sandstone-type regions.

Pisha Sandstone-Type Region	Distribution Region	Main Erosion Types	Main Composite Erosion Types	Average Erosion Modulus/[t/(km^2^·a)]
Bare regions	Kuye River, Huangfuchuan Watershed, Ten Tributaries, etc.	Hydraulic erosion	Hydraulic erosion, wind erosion, freeze–thaw erosion	2.1
Soil-covered regions	Kuye River, Gushanchuan Watershed, Qingshui River, Huangfuchuan Watershed, Hun River, Ten Tributaries, etc.	Hydraulic erosion	Hydraulic erosion, wind erosion, gravity erosion, freeze–thaw erosion	1.5
Sand-covered regions	Kuye River, Gushanchuan Watershed, Ten Tributaries, etc.	Wind erosion	Hydraulic erosion, wind erosion	0.8

## Data Availability

Data are contained within the article.
